# Idiopathic inflammatory myopathy and non-coding RNA

**DOI:** 10.3389/fimmu.2023.1227945

**Published:** 2023-09-06

**Authors:** Yang Yang, Hu GuangXuan, Wan GenMeng, Li MengHuan, Chang Bo, Yi XueJie

**Affiliations:** ^1^School of Kinesiology, Shanghai University of Sport, Shanghai, China; ^2^School of Physical Education, Liaoning Normal University, Dalian, Liaoning, China; ^3^College of Exercise and Health, Shenyang Sport University, Shenyang, China; ^4^Social Science Research Center, Shenyang Sport University, Shenyang, Liaoning, China

**Keywords:** idiopathic inflammatory myopathies, autoimmune disease, non-coding RNAs, biomarker, clinical diagnosis

## Abstract

Idiopathic inflammatory myopathies (IIMs) are common autoimmune diseases that affect skeletal muscle quality and function. The lack of an early diagnosis and treatment can lead to irreversible muscle damage. Non-coding RNAs (ncRNAs) play an important role in inflammatory transfer, muscle regeneration, differentiation, and regulation of specific antibody levels and pain in IIMs. ncRNAs can be detected in blood and hair; therefore, ncRNAs detection has great potential for diagnosing, preventing, and treating IIMs in conjunction with other methods. However, the specific roles and mechanisms underlying the regulation of IIMs and their subtypes remain unclear. Here, we review the mechanisms by which micro RNAs and long non-coding RNA-messenger RNA networks regulate IIMs to provide a basis for ncRNAs use as diagnostic tools and therapeutic targets for IIMs.

## 1 Introduction

Idiopathic inflammatory myopathies (IIMs) are autoimmune diseases characterized by chronic myositis (1). They include polymyositis (PM), dermatomyositis (DM), inclusion body myositis (IBM), immune-mediated necrotizing myopathy (IMNM), antisynthetase syndrome, and overlapping myositis with different subtypes and clinical features (2). Diagnosing IIMs and their subtypes is based on morphological muscle biopsies combined with magnetic resonance imaging, which show peripheral atrophy of the muscle fiber bundles. However, given the damaging nature of muscle biopsies, there is a lack of willingness to test patients in the early stages of the disease when there are no other apparent changes. Using non-invasive or minimally invasive tests would improve early diagnosis rates, suggesting that blood, skin, and hair tests may be important in the future.

Additionally, the current treatment for IIMs mainly involves glucocorticoids and other immunosuppressive agents, which fail to achieve specific therapeutic effects and can even cause adverse effects. Moreover, as some symptoms of non-immune myopathies are similar to those of IIMs, medically induced damage may occur owing to the inappropriate use of immunosuppressive therapy. Therefore, studying the exact pathological mechanisms and searching for diagnostic modalities for microdamaged IIMs and their subtypes are essential for their specific prevention and treatment.

Non-coding RNAs (ncRNAs), including micro RNAs (miRNAs), long non-coding RNAs (lncRNAs), and circular RNAs (circRNAs) (3), were thought to be RNAs without protein-coding capabilities; however, ncRNAs are involved in gene transcriptional regulation processes, such as RNA splicing, processing, editing, and translation (4). MiRNAs can bind to the 3′-untranslated region (5), inhibit messenger RNA (mRNA) expression and translation, and promote degradation after transcription (6). LncRNAs are key regulators of RNA transcription, splicing, and translation (7).

NcRNAs are markers for the development of immune-related diseases, effectively regulating IIM-related symptoms such as the inflammatory response (8) and skeletal muscle production, differentiation (9), and atrophy (10), suggesting that ncRNAs are necessary for regulating the diagnosis, prevention, and treatment of IIMs. However, there is only one review from 2015 on the relationship between miRNAs and IIMs and no description of the possible mechanisms. Therefore, the present paper reports on the progress in using ncRNAs to diagnose, prevent, and treat IIMs to provide a theoretical basis and medical tools for relevant research.

## 2 ncRNA as a possible biomarker

Biomarkers are biochemical indicators that can mark changes or possible changes in body structure or function in different states. They are characterized by their reproducibility, specificity, stability, high sensitivity, and non-invasiveness. In recent years, several studies have considered serum and hair as ideal specimens for biomarker detection. NcRNAs are stably expressed in the serum and multiple tissues of healthy individuals of the same species, are not digested by RNase A, and are less affected by freezing and thawing; however, correlative histological studies show large variations in serum miRNA profiles in patients with IIM. These findings suggest that ncRNAs may be ideal biomarkers for diagnosing IIM and its subgroups (11, 12).

### 2.1 Serum ncRNAs may be associated with IIM and its isoforms

The maturity and progress of high throughput sequencing technology and the rapid accumulation of histological data have prompted an increasing number of studies to combine transcriptomic and other molecular biotechnologies to identify innovative targets and biomarkers. Jiang et al. (13) observed significant changes in miRNA levels after the incubation of exosomes with human aortic endothelial cells for 24 h in healthy controls (HCs) and patients with juvenile dermatomyositis (JDM), suggesting that the analysis and collation of histological results may effectively reveal the serum ncRNAs that may be associated with IIM and its subtypes.

Histological studies have shown that sera from patients with three different diseases (PM, DM, and IBM) at different ages exhibit significant downregulation of miR-1234, -36795p, and -4299 and upregulation of let-7b*, -4310, and -498 compared to those of sera from healthy young men and women. The specific ncRNAs signatures were not the same in the different patient subtypes. In that study, miR-4299 was downregulated in patients with DM, and let-7b* and miR-3907 were upregulated; miRNAs in patients with PM were significantly downregulated and miR-4281 was upregulated (11).

NcRNAs have different specific markers in different IIM subtypes, which may effectively distinguish them; however, there was a large age gap between the subjects in that study and the assay results. It is worth noting that Misunova et al. (12), who focused on recruiting middle-aged patients with IIM, considered age an intervening factor and showed that three miRNAs were significantly upregulated in middle-aged patients with IIM with high activity levels. Three miRNAs were differentially expressed in patients with active DM and PM. In patients with inactive DM, hsa-miR-3648 was upregulated compared to that in patients with inactive PM. In patients with highly active disease, four miRNAs were differentially expressed, while in active and inactive patients, eight miRNAs were differentially expressed (12). This study is the first to reveal that different subtypes of middle-aged patients with IIM have different markers at different disease stages. Therefore, this topic needs to be researched in the future.

Thus, ncRNAs appear to be differentially expressed in different IIM subtypes and between disease courses, suggesting that serum ncRNAs may act as biomarkers of IIM and its subtypes. However, the difficulty of recruiting subjects with IIM has led to an age gap between the selected subjects, and some IIM subtypes, such as DM, may be more likely to occur among adolescents. Future investigators should identify the main age groups for the onset of the different subtypes and focus on recruiting patients in that age group. This may be the key to identifying patients with that disease regarding IIM histology.

### 2.2 Serum ncRNAs as possible IIM markers

The clinical features of IIMs are accompanied by a significant inflammatory response, muscle volume loss, and multiple histological findings (14). NcRNAs changes in the inflammatory response and muscle atrophy also confirm this finding. However, integrating the transcriptomic data from patients with IIM means that thousands of genes and RNAs can be studied simultaneously, revealing a network of interactions associated with IIM at the molecular level. Unfortunately, owing to the current technological limitations, meeting the requirement for biomarker specificity still requires a combination of multiple histological results, further refinement of subtype classification, and biological validation. Therefore, combining ncRNAs that have been tested multiple times histologically and validated by other biological means is more likely to act as an IIM biomarker (**Table 1**).

**Table 1:** Serum ncRNA as a possible IIM marker.

miRNA	Up- or downregulated expression	Target	Tissue/cellular origin	Disease model	Species	Mechanism	Analytical Tool	Ref
miR-7	Down		Plasma	IIM	Human		RT-PCR microarray analysis	(15)
	Down		Serum	DM/PM	Human		Microarray analysis	(16)
miR-21	Up		Serum	DM	Human		RT-PCR	(17)
	Up		Serum	IIM	Human		Microarray analysis	(15)
	Up		Skeletal muscle	Pseudohypertrophic macromyotrophy	Mice	Inflammation, myasthenia gravis	RT-PCR	(18)
miR-146b	Up		Serum/monocytes	DM	Human		RT-PCR	(19)
miR-146a	Down		Serum/monocytes	DM	Human		RT-PCR	(19)
	Down	REG3A	Serum/monocytes/macrophages	DM/cell	Human	Inhibition of macrophage metastasis	RT-PCR Immunomics	(20)
	Down	TRAF6	Serum/skeletal muscle	DM	Rats	Inflammation	RT-PCR Protein blotting	(21)
	Down	IL-17 NF-κB	Serum	IIM	Human	Promotes rhabdomyolysis		(22)

miRNA, micro-RNA; IIM, idiopathic inflammatory myopathy; DM, dermatomyositis; PM, polymyositis; RT-PCR, reverse transcription polymerase chain reaction.

#### 2.2.1 miR-7

A 2018 histological study showed that plasma miR-7 levels were lower in patients with IIM than in those with HCs and in patients with IIM/interstitial lung disease (ILD) than in those without ILD (15). Additionally, Oshikawa et al. (16) observed that serum miR-7 levels were significantly lower in patients with DM, PM, or clinically amyopathic dermatomyositis (CADM) than in those with other autoimmune diseases, including systemic sclerosis, or in HCs. This suggests that miR-7 is an important marker for the effective diagnosis of IIM and correlates with the number of concomitant diseases in IIM. MiR-7 is expressed in immune-related cells, including lymphocytes and fibroblasts, and regulates inflammation development and myoblast growth and differentiation through Toll-like receptor (TLR)4/nuclear factor (NF)-κB (23). This suggests that, although no studies have been conducted directly on TLR4/NF-κB, it has no role in inflammation development. In addition, mir-7 also promotes muscle cell growth and differentiation and improves muscle atrophy (24). Although the role and mechanisms of miR-7 in regulating skeletal muscle in patients with IIM are still unclear, given its important role in skeletal muscle and inflammation and that the plasma miR-7 level varies significantly between patients with IIM, miR-7 may be an important IIM marker and may regulate the level of inflammation and muscle differentiation in IIM.

#### 2.2.2 miR-21

A microarray study of serum samples from patients with DM, PM, SLE, SSC, and HCs showed that miR-21 was significantly upregulated only in the DM group compared to the HCs, and miR-21 levels were positively correlated with serum immunoglobulin levels, a marker reflecting abnormal immune system activation and DM disease (17). The results suggest that miR-21 may be a specific diagnostic marker for DM. In another study, serum miR-21 was only upregulated in some patients with IIM, possibly because the study did not differentiate between subtypes, and this result seems to confirm this inference (15).

Morgoulis et al. (18) demonstrated that miRNAs 21 and 29 activate TLR-7 and TLR-8, promoting the recruitment and complexation of MyD88, and that miR-21 is an important mediator of inflammation and muscle atrophy. IRAK1, IRAK4, and articulin E3 ubiquitin ligase tumor necrosis factor (TNF) receptor-associated factor 6 (TRAF6) ultimately activate NF-κB (25, 26). Additionally, miR-21 activates TLR-7 via the c-Jun N-terminal kinase (JNK) pathway to upregulate the inflammatory factor TNF-α and induce apoptosis in myoblasts (27). The key to miR-21 regulation of inflammation seems to be TLR7; notably, this finding is consistent with the significant changes in TLR-7 in DM disease shown by histological analysis (28).

TNF-α can affect muscle protein hydrolysis by stimulating the NF-κB pathway and ubiquitin-protein vesicle system (22). In the myotonic dystrophy (mdx) pathway, TNF-α affects muscle protein hydrolysis. Inhibition of miR-21 in mdx mice and Duchenne muscular dystrophy fibroblasts improved the upregulation of cell growth factors, PTEN and Sprouty 1, and downregulation of the muscle extracellular matrix markers, type VI collagen (COL) α1 chain COL6A and COL1A1 (29). Interestingly, TLR7 is an upstream factor of PTEN (30), which in turn affects muscle cell growth; PTEN acts as an upstream factor of NF-κB (31), suggesting that miR-21 in IIMs may regulate the inflammatory response through the JNK-TLR7-PTEN/TNF-α-NF-κB pathways to slow muscle growth, which in turn promotes inflammation and a vicious cycle that ultimately leads to muscle and extracellular protein hydrolysis.

#### 2.2.3 miR-146 and miR-23b

Histological analysis of sera from patients with DM showed that miRNAs (23b-3p, 146a-5p, and miR-150-5p) were downregulated and miR-146b-5p was significantly upregulated in the sera and peripheral blood mononuclear cells (PBMCs), with miR-146a-5p being associated with C-reactive protein, an indicator of infection and injury (19). Additionally, the macrophage transfer of REG3A, granulocytes, and serum levels of the inflammatory indicators, interferon (IFN)-γ and interleukin (IL)-17A mRNA, are substantially increased in patients with PM/DM, and miR-146a expression was significantly lower than that in HCs. Cellular assays further demonstrated that miR-146a and IL-17A simultaneously induce REG3A expression, inhibiting macrophage metastasis (20). Yin et al. (21) observed higher levels of creatine kinase (CK) and the inflammatory markers, TRAF6, IL-17, and intercellular adhesion molecule (ICAM)-1, in patients with PM/DM than in HCs.

Genetic assays verified that miR-146a targets TRAF6 and miR-146a mimics significantly downregulate the expression of the inflammatory macrophage infiltration markers, IL-17 and ICAM-1. miR-146a alters the DNA-binding activity of NF-κB in macrophages (32). Interestingly, miR-146a is closely associated with several serum assays of IIMs; miR-146a effectively regulates IL-17 and NF-κB in patients with IIM, while the latter two effectively promote muscle lysis (22). This suggests that miR-146a may be a potent IIM marker and mediate macrophage translocation in IIM to regulate inflammation and thus promote muscle hydrolysis. Based on the possibility that miR-146a may play an important role in IIM, a follow-up study performed a genetic polymorphism analysis of miR-146a; no difference was found in the frequency of the miRNA single nucleotide polymorphism rs2910164 genotype distribution in miR-146a between the HCs and patients with PM/DM. However, the prevalence of muscle weakness and dysphagia was significantly higher in patients with the CC genotype than in those with the C/G or G/G genotypes. The serum detection of the CC genotype in DM patients showed that the miR-146a level significantly decreases, suggesting that such patients are at a higher risk of muscle damage; however, further point mutation techniques are required to reach this conclusion (33).

MiR-23b promotes myoblast proliferation and directly targets the mRNAs encoding cyclins D1 and D2 (34). In contrast to the results of another study (19), no significant differences were found in the expression of miR-23b in the PBMCs from patients with DM and PM compared to HCs (35). However, owing to the small number of subjects in this study, it may be premature to draw this conclusion; a larger population may be necessary. Additionally, plasma hsa-miR-4442 levels in patients with PM/DM were significantly higher than in patients with other autoimmune diseases, and upregulation of the plasma hsa-miR-4442 in patients with PM/DM was positively correlated with the Myositis Intention to Treat Activity index (36).

### 2.3 Skin and hair ncRNAs may serve as IIM markers

Biological fluids provide information mainly in the first few hours after sampling, after which biomarker concentrations vary and, therefore, are unsuitable for assessing chronic interventions. In contrast, hair analysis provides information over several months; thus, it is more suitable for studies on the long-term effects of external factors, such as the environment. MiR-214 levels in the hair of patients with DM are higher than in HCs and patients with scleroderma, suggesting that hair miRNAs may act as independent biomarkers for IIM (37). Additionally, normal juvenile skin tissue shows significant changes in JDM-onset miR-193b, miR-199b-5p, and miR-6653 (38). MiR-223 levels were reduced in the Gottron papules of patients with DM and CADM but not in patients with psoriasis (39). Unfortunately, current studies on IIM appear to be less well-tested in hair and skin.

Thus, histology has revealed many relationships between ncRNAs and IIMs, suggesting that ncRNA may serve as a marker for IIM diagnosis; however, current studies seem to be focused on PM and DM, suggesting that further analysis of patients with different disease courses and subtypes is needed. Additionally, serum miR-7, miR-21, and miR-146a, closely related to several IIM serum indicators, may be valid markers for the diagnosis of IIM, while miR-21 may be a specific diagnostic marker for DM. However, to be used as such, there is still a need to address the relationship between miR-7 and other clinical signs and indicators of the disease (CK, muscle function, and red blood cell indicators), the relationship between miR-7, miR-21, and miR-146 and the development of IIM, the relationship between miR-7 and other clinical symptoms and indicators of the disease (CK, muscle function, and erythrocyte indicators), and the relationship between miR-7, miR-21, and miR-146 and IIM development, as well as the stability of miR-7 in different groups (age, gender) of the disease population.

Histological studies have also shown no significant differences in miR-7 and miR-21, regardless of the subtype and degree of disease activity (12). This question will need to be further validated using biological means at the morphological, protein, and genetic levels. Although associated with this index, there is a lack of experimental evidence that miR-146 modulates the index IIM-related assays; future animal models of muscle inflammation could address this issue. In contrast, hair and skin assays appear more useful than injury-free assays for a deeper investigation of the etiology of non-inherited IIM. Notably, miR-223 seems to suggest a new idea of co-testing serum and hair as a confirmatory test for IIM, and the pairing of the two seems to reflect both the changes in ncRNAs brought about by IIM in real time and the effect of long-term interventions that cause stable changes in ncRNAs.

## 3 Role of ncRNAs in IIM treatment and possible mechanisms

**Figure 1:**
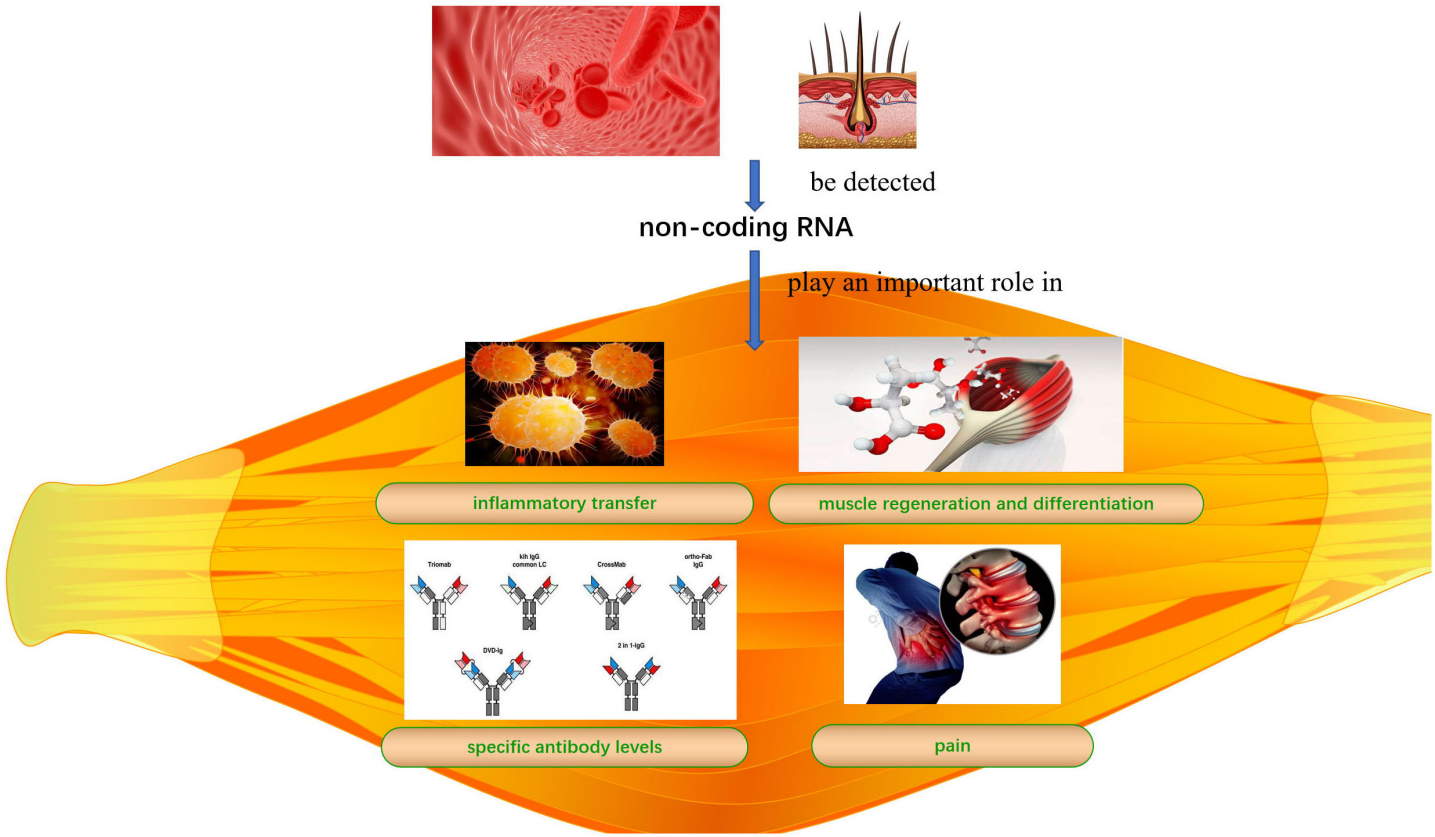
Summary picture.

IIM clinical features mainly include persistent inflammation-induced muscle atrophy and necrosis, culminating in muscle regeneration (40), increased expression of specific antibodies, and severe pain. The first step in muscle regeneration is the inflammatory response, whereby macrophages and lymphocytes invade the area of injury and engulf the necrotic debris to promote a myogenic response. The second step involves satellite cell (SC) activation and differentiation, and the third step involves new muscle fiber maturation and regenerative muscle remodeling and differentiation. All steps are closely related to myogenic regulatory factors (MRFs), such as myogenic factor (Myf)5, Myf6, myostatin, and myogenic cell assay proteins (41, 42). MiRNAs may regulate MRFs through TNF-α and IL-1β, mediating NF-κB, MAPK p2, and other key pathways that inhibit myogenic differentiation into myoblasts/myotubes (43, 44). Therefore, identifying the ncRNAs that effectively regulate the inflammatory response, SC activation and differentiation, myofiber maturation and differentiation, specific antibody expression, and inhibition of muscle pain may be key to treating IIMs with different subtype treatments (**Table 2** and **Figure 1**).

**Table 2:** Role of ncRNA in the treatment of IIMs and possible mechanisms.

miRNA	Up- or downregulated expression	Target	Tissue/cellular origin	Disease model	Species	Mechanism	Analytical Tool	Ref
miR-126	Down		Skeletal muscle	JDM	Human	Inflammation	Microarray analysis	(8)
miR-146a miR-142-3p miR-142-5p miR-455-3p miR-455-5p	Up	NF-κB	Skeletal muscle	patient with DM/mouse	HumanMice	Inflammation	ChIP-seq RT-PCR	(45)
miR-146a miR-146b miR-31 miR-223	Up	NF-κB	Skeletal muscle	patient with DM/mouse	HumanMice	Myasthenia gravis	ChIP-seq RT-PCR	(45)
miR-1 miR-133a miR-133b miR-206	Down		Skeletal muscle cells	patient with DM/mouse	HumanMice	Myoblast differentiation	Microarray analysis RT-PCR	(43)
miR-223	Up	IKK-α	Neutrophils/macrophages	Muscle injury	Mice	Inflammation	RT-PCR	(46, 47)
miR-29a	Up		Myogenic cells	Muscle injury	HumanMice	Myoblast proliferation	RT-PCR	(48)
miR-155	Up	MEF2A	Macrophages	Muscle injury	Mice	Macrophage activation	RT-PCR	(49) (50)
miR-409-3p	Down	CXCR4	Serum/ Skeletal muscle	patient with PM/EAM mouse	HumanMice	Macrophage migration	GSE143845 database RT-PCR	(51)
miR-21	Up	CXCL10	Serum/ Skeletal muscle	patient with PM/PM mouse	HumanMice	Macrophage migration	RT-PCR	(52)
miR-499	Down	AMPK		IIM	Human	Autophagy	Microarray analysis	(53)
miR-1 miR-208b miR-499	Down		Skeletal muscle	PM	Human	Myoblast differentiation and regeneration	Microarray analysis	(53)
miR-133a miR-486	Up		Skeletal muscle	PM	Human	Myoblast differentiation and regeneration	Microarray analysis	(53)
miR-1 miR-133a miR-206 miR-486		Pax7	Skeletal muscle cells	C2C12 cell model	Mice	Satellite cell differentiation	RT-PCR	(54, 55)
miR-1 miR-133a miR-206	Down	Pax7	Skeletal muscle	PM/DM	Human	Satellite cell differentiation	RT-PCR	(56)
miR-486		Pax3 Pax7 MSTN	Skeletal muscle	Normal mouse	Mice	Satellite cell differentiation	Microarray analysis	(57)
miR-486		PTEN FOXO1 MSTN	Skeletal muscle	Normal mouse	Mice	Skeletal muscle growth	Microarray analysis	(57)
miR-195 miR-497		Ccnd2	Myogenic cells and skeletal muscle tissue	Normal	HumanMice	Cell cycle	RT-PCR	(58)
miR-1	Up	HSP70	Skeletal muscle cells	Myasthenia	Mice	Mitochondrial activity	RT-PCR	(58)
miR-133a	Down	nPTB FOXL2	Skeletal muscle cells	Cell differentiation	Mice	Cytoskeleton reorganisation	RT-PCR	(59, 60)

miRNA, micro-RNA; JDM, juvenile dermatomyositis; IIM, idiopathic inflammatory myopathy; DM, dermatomyositis; PM, polymyositis; RT-PCR, reverse transcription polymerase chain reaction; ChIP-seq, chromatin immunoprecipitation sequencing.

### 3.1 Possible mechanisms of inflammatory response triggered by IIMs

Neutrophils are activated after acute muscle injury, and circulating monocytes infiltrate the tissue (61); pro-inflammatory M1 macrophages release appropriate amounts of inflammatory factors shortly after muscle injury to stimulate SC proliferation, while M2 macrophages have anti-inflammatory, pro-myoblast fusion, and myotubular hypertrophy functions (62). Interestingly, during this phase, IIM-specific miRNAs (1, 133a, and 206) are released into the bloodstream; they are stable in a nuclease-rich extracellular environment and protected from RNase-mediated degradation (63), suggesting that ncRNAs may regulate IIM-induced inflammation.

#### 3.1.1 Pro-inflammatory effects of TNF-α

Recent histological and bioinformatic studies showed that plasma exosomal (EXO) TNF-α inducible protein (IFI)6 was highly associated with CD4 T protein infiltration in patients with DM. Gene set enrichment analysis showed that IFI6 was enriched in many immune-related pathways, such as the TNF-α upstream and Janus kinase (JAK)/signal transducer and activator of transcription (STAT) signaling pathways. Applying the miRcode database and DIANA-LncBase to the reverse prediction of miRNAs and lncRNAs yielded 396 miRNA–mRNA and 8769 lncRNA–miRNA interaction pairs. Additionally, compared to HCs, untreated patients with short-course JDM had significantly lower miR-126 and increased serum levels of vascular cell adhesion molecule (VCAM)-1 and TNF-α (8), indicating that DM induces a significant increase in the serum levels of VCAM-1 and TNF-α. In addition, as a co-occurring disease closely related to IIM, multiple lncRNA markers were found in omics tests. Notably, lncRNA-TNF-associated immunomodulatory lncRNA (THRIL) detected in muscle can regulate TNF-α promoters by interacting with heteroribonucleoprotein L (hnRNPL) (64). These studies suggest that TNF-α regulates DM-induced hyperinflammation and that ncRNAs are closely associated with TNF-α expression (1).

Another histological study showed elevated expression of inflammatory miRNAs and myasthenic protein-targeting miRNAs in patients with DM. Chromatin immunoprecipitation sequencing revealed at least one NF-κB consensus element within the promoter/enhancer regions of these miRNAs (45). Elevated NF-κB expression was also observed in muscle mononuclear cell infiltrates from mice and muscle biopsies from patients with severe myositis. The inflammatory factor TNF-κ activates the phosphorylation of inhibitory proteins (IκBs) in the cytoplasm, eliminating the inhibition of NF-κB by IκBs and allowing free NF-κB to translocate to the nucleus to function as a transcription factor, ultimately leading to chronic inflammation (65).

In further experiments on mouse C212 and human skeletal muscle myogenic cells, increased TNF-α inhibited the differentiation of myogenic cells into myoblasts and myotubes by inhibiting miR-1, miR-133a/b, and miR-206 in a nuclear factor NF-κB-mediated manner (43). Additionally, genome-wide analysis revealed that miR-223 is upregulated in neutrophils and macrophages after muscle injury (46, 66). In contrast, miR-223 inhibits IκB kinase (IKK)-α levels in macrophages, thereby inhibiting inflammatory activation of NF-κB and TNF-α production (47) and is involved in chronic inflammation after skeletal muscle injury (67).

The inflammatory infiltration of CD4 T cells plays a crucial role in the occurrence and development of dermatomyositis (DM) (68). The loss of T cell number and function may be an important component of abnormal cytolytic activity in IBM muscle (69). Interestingly, compared with the control group, the expression of lnc-MAF-4 in the PBMCs of MS patients was significantly increased, and the expression level correlated with the annual recurrence rate of MS patients. Further transfection of lnc-MAF-4 activates CD4 T cells in MS patients (48). Compared with the control group, the number of regulatory T cells is reduced in DM patients, the Homeobox D gene cluster antisense growth-related long non-coding RNA (HAGLR) is upregulated, Foxp3 is down-regulated, and RUNX3 protein levels are reduced, but the mRNA levels are not significantly changed. RUNt-associated transcription factor (RUNX3) regulates Foxp3 transcription, while Foxp3 is specifically expressed in Treg cells. Cell experiments showed that HAGLR interference-mediated RUNX3-Foxp3 ultimately affects the Treg cell proportion. *In vivo*, the injection of adv-HAGLR can increase the Treg cell proportion and improve the DM phenotype (70).

NcRNAs and their networks regulate the inflammatory response of IIM and its subtypes; miR-223 may be a key mediator and closely related to the TNF-α-NF-κB pathway. However, owing to the lack of models of skeletal muscle inflammation, future interventions at the cellular level may be required to clarify these mechanisms (68).

#### 3.1.2 Macrophage migration

IIM is accompanied by macrophage activation, inflammatory infiltration, and migration (71), and muscle miRNAs are significantly upregulated in three diseases: PM, DM, and IBM (72). MiR-29a reduces the expression of key basement membrane elements, such as pro-fibronectin-1, collagen-IV, and laminin gamma-1. It also promotes basement membrane dismantling, releases growth factors from the basement membrane, promotes their entry into their receptors, and inhibits macrophage activation (50).

MiR-221, miR-146b, and miR-155 were included in three IIM miRNA studies (43, 49, 72); miR-146 has been previously explored. TLR ligand induction and LPS stimulation upregulate miR-155 expression in macrophages and promote the translation of TNF-α (51), which is expressed by M1 macrophages and exacerbates muscle injury. TNF-α peaks approximately 24 h after injury, which coincides with M1 macrophages invading the muscle tissue (52), suggesting that TNF-α may be an important factor in promoting macrophage migration. After muscle injury, miR-155 expression increases the balance between M1 and M2 macrophage activation, suggesting that TNF-α may be an important factor in promoting macrophage migration. After muscle injury, miR-155 expression increases and mediates muscle growth factor MEF2A expression by regulating the balance between M1 and M2 macrophage activation (73) and targeting the negative regulators of inflammatory signaling pathways, such as SOCS1, JAK, and STAT, to promote muscle regeneration (74). MiR-155 may, thus, be vital in regulating macrophage activation and homeostasis.

Chemokines (CXCRs) are small cytokines or signaling proteins secreted by cells that induce cell-directed chemotaxis. MiR-409-3p is downregulated in patients with PM with elevated CK, TNF-α, and IL-6 levels. MiR-409-3p reduces inflammatory cells, macrophages, TNF-α, and IL-6 in the muscle and serum of EAM mice and suppresses inflammatory responses. Cellular assays demonstrated that miR-409-3p mock transfection reduces macrophage migration and CXCR4 expression and reverses the pro-migratory effect of CXCR4 (75). Thus, miR-409-3p mock transfection reduces macrophage migration and CXCR4 levels and reverses the pro-migratory effect of CXCR4. Skeletal muscle miR-21 expression is upregulated in patients with PM after glucocorticoid treatment compared to that in HCs. Macrophage migration and CXCL10 expression are reduced after miR-21 intervention in rats (76). NcRNAs may regulate macrophage migration through the modulation of chemokines, which in turn mediate disease; however, this has been poorly reported and needs further investigation.

Autophagy regulates phagocytosis and the antigen-presenting functions of macrophages. In addition, it regulates inflammation development and regression by regulating the migration direction of macrophages. A total of 124 upregulated lncRNAs (1392 target genes), 255 downregulated lncRNAs (1867 target genes), 17 upregulated miRNAs (2908 target genes), and 15 downregulated miRNAs (2176 target genes) were found in peripheral neutrophils (i.e., EXO) from patients with DM. Genetic ontological analysis of differentially expressed lncRNAs and mirRNAs showed that they produce IL-6 and IFN-β and promote the proliferation and development of skeletal muscle cells. KEGG analysis indicated that the autophagic pathway, PI3K-Akt, and AMP-activated protein kinase (AMPK) signaling pathways are involved in DM pathogenesis (14). Another histological study showed that the miRNAs in the EXO of patients with DM were involved in its pathogenesis. ENST00000560054.1 induces autophagy in human skeletal muscle cells (77). MiR-499, which is significantly downregulated in patients with IIM, is a key factor in autophagy and inhibits AMPK (54, 78). Additionally, the enhanced cellular autophagy factor KLF in the PBMCs of patients with DM is accompanied by the attenuated expression of miR-206 (79). MiR-499 and miR-206 may regulate inflammation by regulating autophagy, consistent with the histological results (79).

Thus, ncRNAs may be involved in regulating the different roles of macrophages; they can activate macrophages to regulate inflammation and may also mediate macrophage migration by regulating chemokines and autophagy, effectively controlling the development of inflammation and, consequently, muscle growth and differentiation.

### 3.2 Cell differentiation and proliferation

#### 3.2.1 SCs

Abnormally elevated SC activation in patients with IIMs may be influenced by ongoing inflammation, resulting in a reduced pool of SCs that act as a counterbalance and replenishment (53). Activated SCs upregulate MyoD and Myf5 expression (55). MyoD directly activates miR-486 via Ank1.5 (80), miR-206, miR-1, and miR-133a (81).

Compared to HCs, in all samples from patients with IIM, the expressions of miR-1, miR-133a, and miR-133b are downregulated, and miR-206 expression is downregulated in patients with DM (43). MiRNAs (1, 208b, and 499) are downregulated, and miR (133a and 486) are upregulated in muscle samples from patients with PM; the extent of change correlates with PM development (82). This suggests that, although different miRNAs may regulate the muscles of patients with different IIM subtypes, it is mainly miRNAs (1, 133a, 206, and 486).

MiR-1, miR-133a, miR-206, and miR-486 target Pax7 transcripts and promote SC differentiation (56, 80). Notably, MyoD protein expression is upregulated in muscle cells from patients with type 1 ankylosing muscular dystrophy after miR-1 levels are reduced (57). MiR-133a targets the 3′-UTR of serum response factor (SRF). MiR-133a silences this gene to maintain the proliferative SC state (83, 84). Reduced TRAF6 expression and the consequent inhibition of miRNAs (1, 133a, and 206) in patients with PM and DM leads to premature SC differentiation and inhibition of PAX7 expression in the SCs (85). MiR-21, miR-29, and miR-146a are TRAF6 upstream factors, suggesting that they may regulate ncRNAs level and, thus, promote SC differentiation by regulating inflammatory expression; however, this has not yet been reported.

In addition, miR-486 is mainly involved in promoting skeletal muscle growth and hypertrophy by targeting SC differentiation index pairing box gene (PAX)3, PAX7, and MSTN and targeting PTEN, FOXO1, and MSTN (58). Muscle biopsies were taken from different patient populations (inclusion body myositis [IBM] and anti-Jo-1 associated myositis [Jo-1] patient groups). A total of 1287 mRNAs and 1068 mRNAs were differentially expressed in the muscles of the Jo-1 and IBM patients, respectively. Pathway analysis revealed that the Jo-1 and IBM patient groups exhibit co-oxidized phosphorylation and mitochondrial dysfunction. Among them, 16 lncRNAs are differentially expressed in the IBM and Jo-1 groups, including the up-regulated satellite differentiation and regeneration markers H19, lncMyoD, and MALAT1 (77).

Notably, SC differentiation is primed for activation from the resting state, and IIM subtype-anti-3-hydroxy-3-methyl-glutaryl-coenzyme A reductase (HMGCR) antibody-positive myopathy shows elevated Bcl-2 (86), as observed by Miao et al. (87). MiR-195a-5p and miR-125b-1-3p can induce apoptosis in muscle cells by inducing the *Bcl-2* gene. Cell cycle arrest in resting SCs is controlled by miR-195 and miR-497 (88). MiR-195/497 targets the transcripts of genes encoding the cell cycle protein family (Ccnd2), cell division cycle family (Cdc25a and Cdc25b), and cell cycle activators that are expressed in quiescent SCs and protect them from cell cycle effects (89).

The degree of decreased SC differentiation in patients with PM may, thus, be mediated by miRNAs (1, 133a, 206, and 486) mediating Pax7. Although no studies on the ncRNAs activation of SCs have been reported, miR-195a may be an important target.

#### 3.2.2 Role of ncRNAs in muscle cell maturation and differentiation in IIM and its mechanism of action

Few studies have directly reported muscle cell maturation and differentiation in patients with IIM, but the associated changes in miRNA levels seem to reveal some phenomena. Several studies have reported significantly lower miR-1 and miR-133 levels in the serum and EXO of patients with IMM.

MiR-1 enhances protein 1 synthesis and ATP production in myoblast mitochondria without increasing the mitochondrial DNA copy number or transcription (44). In contrast, aberrant mitochondrial proliferation leads to excess oxygen radicals and enhances senescence and inflammation. Kukreti et al. (90) observed that miR-1 binds to and reduces heat shock protein 70 downregulation of p-Akt, thereby enhancing mitochondrial Foxo3 nuclear activity and inhibiting the upregulation of the atrophy marker muscle finger protein atrogin-1 (90). This suggests that the regulatory effect of miR-1 on mitochondria, important organelles that provide the skeletal muscle with the energy required to exercise, may directly affect mitochondrial oxidation, enhancing their function and quality rather than regulating their quantity. MiR-1 is reduced in the serum after prednisolone treatment in patients with PM/DM compared with HCs (59). Thus, reducing miR-1 expression may be an important tool for effectively treating IIMs.

MiR-133a is of the same origin as miR-1 and is expressed by SRF (60) and mTOR complex 1 via MyoD (91). MiR-133 upregulates the expression of the pro-myogenic transcription factors MEF2C and MAML1 (92). In promoting differentiation, miR-133a also targets the neuronal polypyrimidine tract-building protein and the Forkhead box L2 transcription factor (93, 94), which regulates hypertrophic and actin cytoskeletal reorganization. In contrast to miR-133a, miR-133b has little obvious targeting; however, the targets of miR-133a and miR-133b overlapped greatly (95, 96).

Additionally, miR-23a was downregulated in patients with IIM, whereas muscle miR-1, miR-208b, and miR-499 were downregulated in patients with PM. MiR-1 and miR-208 are key factors that regulate myofiber regeneration rate, myofiber area, and myosin heavy chain gene expression (84, 97, 98). MiR-208b and miR-499 both downregulate miR-208 binding, constituting a negative loop (82, 99) and are associated with the switch between type II and I myofibers (100, 101). MiR-499b converts muscle from the glycolytic to oxidative type by directly inhibiting the translation of transcriptional repressors, Sox3, Purβ, Sp1, and Hp-β (102). Additionally, miR-23a protects slow fibers from fast fiber-specific gene expression (103). Although no studies on IIM muscle fiber-type conversion have been reported, NC may play an important role.

## 4 ncRNAs role and mechanism in melanoma differentiation-associated 5 regulation

Anti-MDA5 is one of the five antibodies associated with DM. Histological screening showed that 38 miRNAs are upregulated and 21 are downregulated in exosomes from patients with pre-treatment DM complicated by ILDs compared to EXOs from HCs. Fifty-one miRNAs are upregulated and 33 are downregulated in EXOs from patients with DM without ILDs compared to those from patients with pre-treatment DM complicated by ILDs. The specific ncRNAs are not the same in different subtypes with different comorbidities; miRNA regulation in patients with DM with specific antibodies may be crucial for DM treatment. MiR-4488 and hsa-miR-1228-5p are the most common differentially expressed miRNAs. Analysis of the PPI network suggested that DExD-decapping enzymes, 39 B and MDM2, may be involved in the DM-ILD-MDA5 Ab(+) mechanism (28). The PA analysis of 26 pairs of differentially expressed miRNAs in patients with anti-MDA5-associated compared to HCs was performed by IFN-β, TLR3, TLR7, TLR9, and PU1 as upstream factors and were closely associated with type I IFN signaling and the C-C motif ligand 2 pathway (104). MiR-150-5p is downregulated in patients with DM with anti-MDA5 antibodies compared to those without antibodies, suggesting that miR-150-5p is a detectable antibody that aids MDA5 (19).

MDA5 expression is upregulated in yellow croaker (*Larimichthys polyactis*) following stimulation with the TLR3 agonist poly(I:C). Bioinformatic and genetic assays indicated the inhibitory effect of miR-203 on MDA5 in yellow croaker (105). MDA5 is a putative target gene for miR-145-5p, and miR-145 is more effective than the miR-145-5p mimic in inhibiting MDA5 and regulating the viral pattern recognition receptor signal RLR (RIG-I-like receptor); the effect is dose- and time-dependent (106). Infectious bursal disease virus (IBDV) infection or poly(I:C) treatment increases the expression of GATA-binding protein 3 (GATA3), a master regulator of TH2 cell differentiation that directly binds to its promoter to promote miR-155-5p expression. Cellular assays confirmed that MDA5, TBK1, and IRF7 are required for GATA3 expression in poly(I:C) host cells. IBDV induces GATA3 expression via the MDA5-TBK1-IRF7 signaling pathway, inhibiting GATA3-mediated gga-miR-155-5p expression during IBDV replication (107). TLR3 appears central to regulating disease in patients with DM. Although there are no reported direct relationships between MDA5 antibodies and ncRNAs in patients with IMNM, it may have a therapeutic effect by regulating ncRNAs.

### 4.1 Role of ncRNAs in regulating other antibodies and mechanisms

The subgrouping of patients with DM according to ILD and anti-Jo-1 antibody status revealed different patterns of lncRNA expression. IFN1 transduction is the most significantly dysregulated pathway in the DM subgroup. Bioinformatic predictions suggest that linc-DGCR6-1 may regulate the IFN1 induction of USP18, which is highly expressed in the perifascial region of muscle fibers in patients with DM (108). Notably, miR-146a expression was confounded with the type 1 IFN pathway by the level of leukocyte infiltration into muscle tissue (49). The isolation and processing of the fascia of the muscle fibers may have been critical during the experiment, and this part of the histological assay should not be overlooked. IFN transmission and antiviral response pathways are upregulated in patients with PM and DM compared to HCs. Anti-Jo1 autoantibody-positive subgroups show more pronounced pre-activated IFN conductance. miR-96-5p is upregulated, and its mRNA targets (adenosine kinase [ADK], CD28, and SLC4A10) are downregulated in these patients, as verified via a polymerase chain reaction. Transfection of human skeletal myocytes with miR-96-5p mimics resulted in ADK downregulation (109).

Antibodies against an enzyme in the endoplasmic reticulum-HMGCR are found in biopsy specimens from patients with IMNM and much less frequently in other muscle diseases. Serum CK (s-CK), high mobility group box (HMGB)1, and cluster of differentiation (CD)163 levels are higher in patients than in HCs, and miR-381 is downregulated. Mice show decreased muscle inflammation and CD163, s-CK, HMGB1, IL-17, and ICAM-1 expression in the anti-IL-17 and anti-HMGB1 groups compared to those in the EAM model group. MiR-381 mimics transfected into macrophages reduce inflammatory macrophage migration and decrease the expression of HMGB1, IL-17, and ICAM-1 (110).

In summary, although current antibody research on IIMs has focused more on histology, this seems to reflect that different antibodies may play different roles; HMGCR, for example, not only regulates inflammatory processes but may also regulate the activation of muscle SCs.

## 5 ncRNAs may be a therapeutic target for pain relief

In recent years, pain has been recognized as a disease; IIM is accompanied by severe pain affecting quality of life. The active regulation of neurological genes plays an important role in developing and maintaining inflammatory pain in skeletal muscles. Complete Freund’s adjuvant (CFA) attracts macrophages and other cells to the injection site for the sustained release of antigens, thereby enhancing the immune response; however, it produces inflammation and damage at the inoculation site, causing severe pain. Unilateral injection of CFA into the rat occlusal muscle results in significant downregulation of mature miRNA in the ipsilateral mandibular branch (V3) of the trigeminal ganglion (TG) T. No significant changes were observed in the ipsilateral V3 in the TG or saline control groups. The time to return to the differences in the downregulated miRNAs was equal to that in naïve animals varied by miRNAs but was at least four days (111). Both muscle and paw mechanical nociceptive hypersensitivity caused by carrageenan-induced muscle inflammation is reduced when artificially produced miR-acid sensing ion channel (ASIC)3 is delivered *in vivo* to the muscles of mice. Levels of ASIC3 mRNA in the DRG and proteins in the muscle are reduced. To investigate the possibility that ASIC1 plays a regulatory role, the expressions of ASIC1a and ASIC3 in CHO-K1 cells were down-regulated by the cell knockdown technique. After re-administration of miR-ASIC3, the amplitude of acidic Ph-induced current decreased significantly (112).

Therefore, targeted modulation of ncRNAs may effectively reduce IIM-associated pain, and the use of artificial miRNAs may give credence to this idea.

## 6 ncRNAs may mediate exercise to alleviate IIM symptoms

Exercise effectively improves the inflammatory response and inflammation-induced muscle atrophy in skeletal muscles. However, its efficacy in alleviating IIM symptoms is not yet well reported.

Exercise was found to alter 39 miRNAs in IIMs that target transcripts involved in inflammatory processes and muscle atrophy. Analysis revealed increased expression of muscle function mitochondrial proteins (AK3 and HIBADH) and decreased expression of the NF-κB regulator IKKB, closely associated with increased miR-196b levels. This suggests that exercise may mediate the effective regulation of IIMs via miRNAs (113). Notably, neither six-month training (in patients with IIM) nor *in vitro* palmitate treatment modulated myotubular muscle decline compared to that in HCs. However, miR-133a, miR-133b, and miR-1, which are associated with skeletal muscle differentiation, were significantly altered and negatively correlated with the expression of lipid metabolism-related FOx, triacylglycerol, and OXPHOS complex-V in myotubes. Myotubular miR-133a and miR-133b are associated with disease activity and fasting glucose *in vivo* (114). This suggests that, although the phenotype was not significantly altered, post-exercise changes in these three miRNAs are critical for SC differentiation and muscle remodeling.

Notably, miR-1 represses the expression of genes that control muscle structure and function (115). These targets include chlorine voltage-gated channel 3, a key factor that regulates the transition from fibroblasts to myofibroblasts (116). The measurement of the IIM function after exercise should be the next step in the examination. As resistance exercise is moderately sustained and does not stimulate the muscles, it should be considered for future inclusion. Unfortunately, stretching during centrifugal exercises can cause muscle damage, pain, inflammation, and elevated serum CK. Therefore, high-intensity interval training methods to increase strength and muscle mass may be the key to this problem. The muscle factors, miR-133, miR-133a, and miR-206b, which are closely associated with IIMs, are significantly regulated by exercise (117), suggesting that the stress response of miRNAs may be part of the adaptive response and muscle memory.

Additionally, post-exercise mRNA burst trajectories are strongly coupled with miRNA trajectories, promoting effective gene expression (118). Long-term training can target histone-modifying enzymes (histone deacetylase [HDAC] and sirtuins). The accumulation of miRNA bursts can lead to pro-adaptive memory (119, 120). HDAC4 activity is regulated by miR-206 (121). HDAC4 interacts with muscle cell-specific enhancer factor 2C to inhibit its functional expression (91, 122). However, epigenetic correlation has yet to be reported during the exercise of patients with IIM, and future studies in this area should be conducted.

## 7 Conclusion

Inflammation of the skeletal muscle can seriously affect the quality of life. While the key to treating IIM lies in its early diagnosis, numerous studies have shown that the ncRNAs network may be a biomarker for early diagnosis and may be involved in post-inflammatory muscle regeneration, alleviating disease-associated pain.

However, there are still limitations: (1) current studies on IIM diagnosis are focused on histology and are relatively lacking in animal models; as it is difficult to identify such models, obtaining cells from patients for culture will play an important role in further validating the mechanism of IIM. The degree of development of different populations and diseases is not currently clear, and non-congenital factors, such as overexertion, lead to IIM. There are no clear reports yet on the role of environmental factors in relation to miRNA; perhaps, more studies have focused on miRNAs because lncRNAs were discovered later (2). There are few studies on the disease and stages of muscle maturation and the relationship of IIM with ncRNAs in the later stages of treatment and rehabilitation and applying ncRNAs in therapy. (3) The influence of the intensity, form, and duration of exercise on ncRNAs is unclear. With modern molecular biology technologies, such as next-generation sequencing, more molecular regulatory mechanisms of ncRNAs and ncRNA-targeted drugs will be discovered. These findings will provide new strategies for the clinical diagnosis and targeted therapy of IIMs.

## Author contributions

YY was responsible for topic selection and main content writing. YX and CB guided and revised the full text. HG assisted YY in literature collection. WG and LM were responsible for graphic production.

## Glossary

**Table:**

ADK	adenosine kinase
ASIC	acid sensing ion channel
AMPK	AMP-activated protein kinase
CADM	clinically myopathic dermatomyositis
CD	cluster of differentiation
CFA	complete Freund’s adjuvant
circRNA	circular RNA
CK	creatine kinase
COL	collagen
CXCR	chemokine
DM	dermatomyositis
EXO	exosomal
HCs	healthy controls
HDAC	histone deacetylase
HMGB	high mobility group box
HMGCR	3-hydroxy-3-methyl-glutaryl-coenzyme A reductase
IBDV	infectious bursal disease virus
IBM	inclusion body myositis
ICAM	intercellular adhesion molecule
IFI	inducible protein
IFN	interferon
IIM	idiopathic inflammatory myopathies
IKK	IκB kinase
IL	interleukin
ILD	interstitial lung disease
IMNM	immune-mediated necrotizing myopathy
JAK	Janus kinase
JDM	juvenile dermatomyositis
JNK	c-Jun N-terminal kinase
lncRNA	long non-coding RNA
MDA	melanoma differentiation-associated
mdx	myotonic dystrophy
MRF	myogenic regulatory factor
mRNA	messenger RNA
miRNA	micro RNA
Myf	myogenic factor
ncRNA	non-coding RNA
NF	nuclear factor
PAX	pairing box gene
PBMCs	peripheral blood mononuclear cells
PCR	polymerase chain reaction
PM	polymyositis
SC	satellite cell
S-CK	serum CK
SRF	serum response factor
STAT	signal transducer and activator of transcription
TLR	Toll-like receptor
TG	trigeminal ganglion
TNF	tumor necrosis factor
TRAF6	TNF receptor-associated factor 6
V3	mandibular branch
VCM	vascular cell adhesion molecule
